# Design and Dynamic Locomotion Control of Quadruped Robot with Perception-Less Terrain Adaptation

**DOI:** 10.34133/2022/9816495

**Published:** 2022-02-22

**Authors:** Lei Wang, Libo Meng, Ru Kang, Botao Liu, Sai Gu, Zhihao Zhang, Fei Meng, Aiguo Ming

**Affiliations:** ^1^Intelligent Robotics Institute, School of Mechatronical Engineering, Beijing Institute of Technology, Beijing 100081, China; ^2^Beijing Advanced Innovation Center for Intelligent Robots and Systems, Beijing Institute of Technology, Beijing 100081, China; ^3^Department of Mechanical Engineering and Intelligent Systems, The University of Electro-Communications, Tokyo 182-8585, Japan

## Abstract

In this paper, a parallel quadrupedal robot was designed that is capable of versatile dynamic locomotion and perception-less terrain adaptation. Firstly, a quadrupedal robot with a symmetric legs and a powerful actuator was implemented for highly dynamic movement. Then, a fast and reliable method based on generalized least square was proposed for estimating the terrain parameters by fusing the body, leg, and contact information. On the basis of virtual model control (VMC) with the quadratic program (QP) method, the optimal foot force for terrain adaptation was achieved. Finally, the results obtained by simulation and indoor and outdoor experiments demonstrate that the robot can achieve a robust and versatile dynamic locomotion on uneven terrain, and the rejection of disturbances is reliable, which proves the effectiveness and robustness of this proposed method.

## 1. Introduction

Legged animals, such as cats, can perform versatile movements on diverse terrain, owing to their structure and cooperative motion. Imparting this ability to an artificial robot is an enormous challenge in the field of robotics. The design of legged robots has benefited from the observation of animal structure. Quadruped robots have the natural advantages of trafficability and agility on complex outdoor terrain compared with wheeled robots [1]. In recent years, many advanced quadruped robots have been achieved in terms of dynamic motion and stability, such as BigDog [2], StarIETH [3], HyQ [4], ANYmal [5], MIT Cheetah [6–8], Jueying [9], BQR [10], and Minitaur [11]. To realize the more practical tasks of quadruped robots, such as material transportation and rescue in complex environments, it is important to investigate the leg structure and locomotion stability on uneven terrain.

Dynamic locomotion is preliminarily determined by the ground reaction force (GRF), which can be characterized by the leg structure and actuators. Generally, series [6] and parallel [12, 13] are the main two leg topologies, and series articulated robots have a big leg motion range. However, one of the joints may be subjected to a heavy load or need more higher joint velocity for fast dynamic movement. The parallel leg is advantageous for force production but may waste energy when the leg swings back and forth. Torque density may be a significant metric for actuators design for dynamic robot. Hydraulic actuation can provide a powerful GRF, but it is not ideal for use in everyday situations. Recently, an electric actuation method combining a high-torque density electromagnetic actuator and low-ratio gear was proposed and can effectively balance the requirements for transparent transmission and high-torque generation. Inspired by this, a high-torque density actuator was designed and assembled using a custom motor and two-stage planetary gearbox.

To improve the trafficability on uneven terrain, accurate terrain estimation and robot adaptation are necessary. The walking surface modeling method has been proposed to approximate the local slope for each walking position with the absence of vision [14], and the least squares problem has been solved using the pseudoinverse. The slope angle was calculated by considering the weighted average of the inertial measurement unit (IMU) information, and the support plane was calculated using least squares estimation [15]. The previously proposed locomotion adaptation method [16] consists of the adaption of control frame, trunk orientation, stance legs, and swing leg motion. The spatial positions of three feet were selected to fit the support plane based on the vertical relationship between in-plane vector and normal vector [17]. Additionally, some studies have used the straight slope between the front and back footholds to approximate the inclination of the support plane directly [18]. These two methods are convenient to calculate, but the solution accuracy is poor.

To enhance the dynamic motion ability and controllability of quadruped robot, this study implemented parallel leg structures with symmetrical rods which match with the low reduction ratio planetary reducer to improve the back-drivability. Then, aiming at traffic capacity in uneven terrain, the locomotion control framework based on VMC [19] was established and mainly includes GRFs optimal allocation based on the QP method and a full terrain adaptation method without perception. The proposed terrain adaptation method uses the generalized least square method to estimate the space supporting plane only by fusing the trunk orientation and joint encoder information without additional perceptual or visual support and then adapts the support plane to achieve the balance of the robot through the adaption of body state and swing leg movement. The proposed method achieves better versatility, reliability, and accuracy results. In addition, it was validated through simulation and various experiments on the prototype of our BQR-2 quadruped robot.

The rest of this paper is organized as follows. The leg topology and actuator design are introduced in Section 2. The modeling and control framework are presented in Section 3.  Section 4 presents the estimating of supporting plane and online adaption. The simulation and experimental results are discussed in Section 5. The conclusions drawn from this study and directions of future work are discussed in Section 6.

## 2. Robot Design with Symmetric Leg

**Figure 1:**
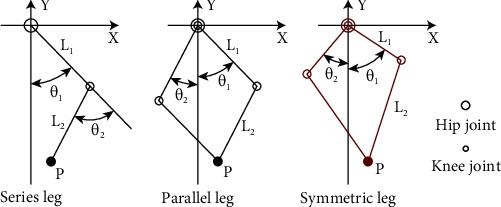
The three kinds of leg topology recruitment.

**Figure 2:**
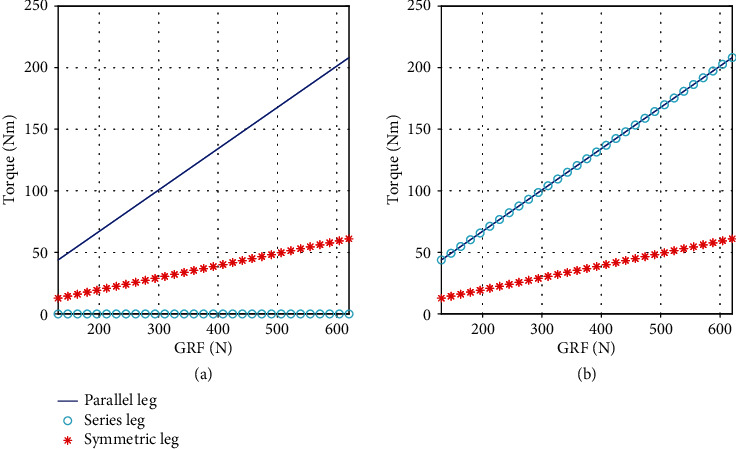
The maximum joint torque of three kinds of leg with GRF. (a) Left and (b) right plots are the maximum torque for hip joint and knee joint of three topology leg.

**Figure 3:**
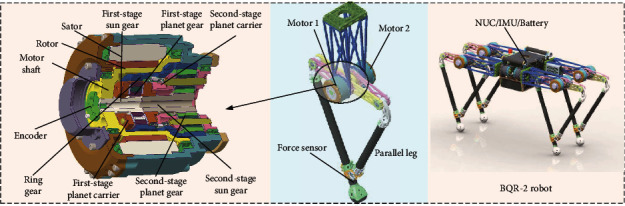
Actuator, leg and robot design with a symmetric topology, and the actuator designed with a custom two-stage planetary reducer.

Force production plays a crucial role in legged robot dynamic locomotion due to repeated impact and continuous high force interaction with ground. In this study, we mainly focused on three leg topology and actuator designs because the force production is closely related with leg Jacobian. This was compared within the leg topology of series [6], parallel [12], and symmetric [20], as shown in Figure 1. Only the flexion motion of hip (*θ*_1_) and knee (*θ*_2_) joint are presented in this paper, *L*_1_ and *L*_2_ are the length of thigh and shank leg. *L*_1_ is equal to *L*_2_ in the condition of series and parallel leg, and *L*_2_ is double of *L*_1_ in the condition of symmetric leg according to parameter from above-mentioned robot.  Figure 2 shows the relationship of GRF and maximal joint torque. The data comes from a numerical simulation with MATLAB; in this case, a constant GRF was inputted every time in stance phase; then a set of joint torque can be got by *τ* = *J*_*i*_^*T*^*f*, and we selected the maximum value to compare. *J*_*i*_^*T*^ are the Jacobian of three legs. With the increasing of GRF, the other group of joint torque data can be collected. Obviously, for the series leg, the hip joint torque is zero, and the knee joint torque is equal to the double joint torque of the symmetric leg. The two joint torques of the symmetric leg are equal and are small. Generally, in terms of leg topology, the symmetric leg requires a small torque source, which means that it can produce larger GRF with the same actuator. Additionally, actuator torque density is a metric we focused. Hence, a custom high torque motor with a two-stage planetary reducer was designed to improving torque density. As shown in Figure 3, the first-stage sun gear linked with the motor shaft; both the first-stage and second-stage planet carriers are connected with the part of ring gear. Obviously, most part of the two-stage planetary reducer is within the motor to ensure a small size. Furthermore, the reduction ratio of 17.4 was selected considering the trade-off between torque density and leg transparency.

Finally, a BQR-2 robot, as shown in Figure 3, is designed with a characteristic of large force production. To estimate the state of robot, a high precision encoder (EBI 1135) was equipped with the motor, a six-axis inertial measurement unit (IMU-MTi100) was mounted at the center of trunk, and the three-dimensional force sensor was located under each foot to measure the GRF. In this robot, NUC was the upper computer and the Elmo Motor Driver was the lower controller, and the control period is 1 kHz over EtherCAT. The robot mass is about 40 kg, and the leg length is 0.6 m. To improving the locomotion performance, the main mass is located in the robot body base, and the lower leg was built with a carbon. As shown in Figure 3, the controller, driver, and battery were all located in the center of this robot.

## 3. Locomotion Control with Optimization of GRF

**Figure 4:**
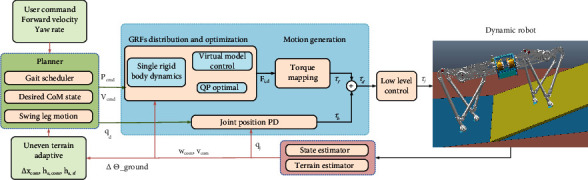
Block diagram of our framework.

This section introduces the proposed locomotion control framework, which includes a motion planner, VMC with QP optimization, a state and ground estimator, and a low-level controller, as shown in Figure 4. The control target is to adjust the position and orientation of robot base according to the robot state, which can be achieved by regulating the GRFs through QP optimization at the standing phase. The constraints imposed by the friction cone and joint motor were considered in the QP optimization. Finally, the optimal solution was mapped into the joint torques by Jacobian. During this process, a fast method for estimating the full terrain information is proposed to balance the robot posture.

### 3.1. Dynamic Model

**Figure 5:**
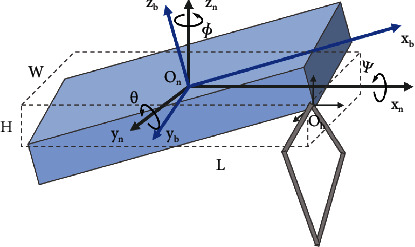
Illustration of coordinate systems and the single rigid body model.

Figure 5 illustrates the coordinate of robot systems. Here, {*O*_*n*_ − *x*_*n*_*y*_*n*_*z*_*n*_} and {*O*_*b*_ − *x*_*b*_*y*_*b*_*z*_*b*_} represent the world and body coordinates, respectively; *ψ*, *θ*, *ϕ* are the roll, pitch, and yaw angle; and *R* is the rotation matrix of the body frame expressed in the inertial frame.

(1) $$\begin{array}{c} R=R_{z}\left(\psi \right)R_{y}\left(\theta \right)R_{x}\left(\phi \right). \end{array}$$

For highly dynamic locomotion, the lower leg rod was designed with carbon to reduce the leg mass and inertial, and the actuator is located near the base. So the most mass is concentrated in the robot body, and for simplicity, in this paper, the robot leg can be as massless. It is assumed that GRF is the only external force acting on the feet, and the robot pitch and roll velocity are small. Then, the GRFs can be formulated as a function of the linear acceleration and the angular acceleration of the body base. The dynamic model can be expressed as follows:

(2) $$\begin{array}{c} \underset{A}{\underset{⏟}{\left[\begin{array}{ccc} I_{3} & \cdots & I_{3}\\ r_{1}\times & \cdots & r_{i}\times \end{array}\right]}}\underset{x}{\underset{⏟}{\left[\begin{array}{c} F_{\text{leg},1}\\ \cdots \\ F_{\text{leg},i} \end{array}\right]}}=\underset{b}{\underset{⏟}{\left[\begin{array}{c} m\left({\ddot{x}}_{\text{com}}^{d}+g\right)\\ I_{g}{\dot{w}}_{b}^{d} \end{array}\right]}}, \end{array}$$

where *m* and *I*_*g*_ are the robot mass and inertia; *g* is the gravity; r_*i*_ × ∈ℝ^3×3^ is the relative position matrix of the *i*^th^ leg; and $\dot{\omega },{\ddot{x}}_{\text{com}}^{d}\in {\mathbb{R}}^{3}$ are the desired angular acceleration of the robot's base and the acceleration of CoM.

In the proposed control framework, the gait can be defined by a finite state machine, which is time- and event-based. The control objectives are the desired trajectories of the CoM position, orientation of trunk, and swing feet trajectories. To reduce the impact disturbance, the cubic spline interpolation method can be used to plan the foot locomotion to achieve a smooth trajectory.

### 3.2. Motion Control

To achieve robust and efficiency locomotion, the VMC was adopted as a control strategy based on the current and target state.

(3) $$\begin{array}{c} \left[\begin{array}{c} F_{c,d}\\ \tau_{b,d} \end{array}\right]=\left[\begin{array}{c} K_{p,p}\left(p_{c,d}-p_{c}\right)+K_{d,p}\left({\dot{p}}_{c,d}-{\dot{p}}_{c}\right)\\ K_{p,w}\left(q_{b,d}-q\right)+K_{d,w}\left(w_{b,d}-w\right) \end{array}\right], \end{array}$$

where *F*_*c*,*d*_ and *τ*_*b*,*d*_ are the virtual force and torque acting on CoM; *K*_*p*,*p*_, *K*_*p*,*w*_ and *K*_*d*,*p*_, *K*_*d*,*w*_ represent virtual proportional gain and derivative gain, respectively; *p*_*c*,*d*_ and ${\dot{p}}_{c,d}$ are the desired position and velocity, respectively; *p*_*c*_ and ${\dot{p}}_{c}$ are the actual position and velocity, respectively; *q*_*b*,*d*_ and *w*_*b*,*d*_ are the desired trunk angle and angular velocity, respectively; and *q* and *w* are the actual trunk angle and angular velocity, respectively.

Combined with Equations (2) and (3), the solution of the GRF can be transformed as an optimization problem, as

(4) $$\begin{array}{c} \begin{array}{c} f^{*}=\underset{f\in {\mathbb{R}}^{6}}{\min}{\left(\text{Af}-b\right)}^{Τ}\text{S}\left(\text{Af}-b\right)+\alpha f^{Τ}\text{Wf}\\ \text{s}.\text{t}.\;-\mu F_{leg,\text{i}}^{n}\leq F_{\text{leg},\text{i}}^{t}\leq \mu F_{\text{leg},\text{i}}^{n}, \end{array} \end{array}$$

where *S*, *W* ∈ ℝ^6×6^ are the weight matrices, *α* ∈ ℝ is the secondary objective, and *μ* is the friction coefficient.

This problem can be solved by QP optimization, and this QP problem can be resolved by 0.15 ms. To improving motion tracking, some basic physical constraints, such as the friction cone and the unidirectionality of the leg output force in the *z* direction, should be satisfied. The constraints of *i*th GRFs are given as

(5) $$\begin{array}{c} \left\{\begin{array}{c} f_{i,x}^{2}\leq \mu f_{i,z}^{2}\\ f_{i,y}^{2}\leq \mu f_{i,z}^{2}\\ f_{\min}\leq f_{i,z}\leq f_{\max} \end{array}\right.⟶\left\{\begin{array}{c} -\infty \leq f_{i,x}-\frac{\sqrt{2}}{2}\mu f_{i,z}\leq 0,\\ \infty \geq f_{i,x}+\frac{\sqrt{2}}{2}\mu f_{i,z}\geq 0,\\ -\infty \leq f_{i,y}-\frac{\sqrt{2}}{2}\mu f_{i,z}\leq 0,\\ \infty \geq f_{i,y}+\frac{\sqrt{2}}{2}\mu f_{i,z}\geq 0,\\ f_{\min}\leq f_{i,z}\leq f_{\max}. \end{array}\right. \end{array}$$

As shown in Figure 4, the command torque in stance leg *τ*_*f*_ can be mapping by leg Jacobian from GRFs based on Equation (4), and the feedback *τ*_*b*_ can be obtained through the joint PD as the swing phase command.

## 4. Adaptation Locomotion on Slope Terrain

For legged robots, adaptive control is an important requirement of adaptive locomotion performance in complex terrain. The most important adaptive control components are the estimation of the unknown terrain environment and the corresponding online adjustment strategy.

### 4.1. Estimation of Supporting Plane

In the accuracy of state estimation during the robot's walking movement, a method for full terrain estimation based on the generalized least squares method is proposed to calculate the space sup-porting plane. In this method, the foot positions in the world coordinates are obtained by fusing the trunk orientation information from the IMU and the joint encoder information without additional perception. The walking surface can be modeled as a space plane [8, 16], as follows: *Ax* + *By* + *Cz* + 1 = 0. The terrain estimated was a virtual plane decided by foot contact point; therefore, the robot can negotiate a terrain with moderate dents and bumps.

The distance from each foot point to the target plane *P*_*i*_(*x*_*i*_, *y*_*i*_, *z*_*i*_)^*T*^ can be solved by constructing the minimum problem of the sum of squares of the distances to obtain the optimal support plane, as follows:

(6) $$\begin{array}{c} f\left(P_{i},A,B,C\right)=\min\overset{4}{\underset{i}{\sum }}d_{i}^{2}\left(P_{i},A,B,C\right), \end{array}$$

(7) $$\begin{array}{c} \frac{\partial f}{\partial A}=\frac{\partial f}{\partial B}=\frac{\partial f}{\partial C}=0, \end{array}$$

where *d*_*i*_ = *nP*_*i*_ + 1 is the distance from the *i*th foot to the desired plane and *n* = (*A*; *B*; *C*) is the normal vector of walking surface plane.

The generalized least squares problem is actually a minimization problem, and the solution objective is to determine a set of optimal coefficients by minimizing the error between measured value and the model value. This problem can be solved by finding the poles of the error function, as expressed by Equation (7). Then, the inclination angles of the foot support plane can be calculated according to the relationship between the normal vector and the reference plane, which is the basis of the subsequent adaptive control.

### 4.2. Online Adaption in Slope Terrain

**Figure 6:**
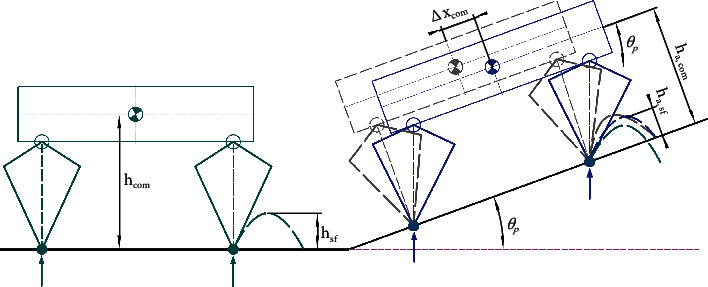
Adaptive strategy in uneven terrain. The green model represents the equilibrium state on the flat ground, the gray model is the transition state after only rotating the tilt angle, and the blue model is the adaptive state on the slope.

To achieve stable locomotion, it is necessary to adjust the robot's posture online according to the estimated terrain information. This subsection introduces the control strategy for the robot system's adaptation to uneven terrain. Here, pitch direction attitude adjustment is considered an example, and the roll direction is the same. On flat terrain, the desired equilibrium state of the robot is as follows: the *Z* axis of the body base is aligned with gravity, the *X* axis is perpendicular to gravity, and constant body height is maintained. On a slope, however, the body base should adapt according to the slope to achieve a new balanced state. As shown in Figure 6, the proposed adaptive method mainly includes the adjustment of the trunk orientation and the adaptation of the swing leg trajectory.

As shown in Figure 6, to maintain a balance when the robot is on the slope, the balance posture on the horizontal plane should be rotated to obtain the posture shown in the gray model. This process only realizes the adjustment of the trunk direction. According to the stability criterion, the net force and moment acting on the robot must be zero. Additionally, the supporting leg (virtual dotted line with the same color) must be adjusted to the position parallel to the gravity direction due to the gravity disturbance in this state. Additionally, this adjustment makes the CoM position move the offset Δ*x*_com_ in the upward direction of slope, and the leg length correspondingly increases. To ensure the adjustable range of the legs in unknown future motion, the CoM position should be adjusted along the gravity direction. Through simplification, it is concluded that, on the slope with *θ*_*p*_, the adjustment in the upward direction and vertical direction of the slope can be calculated as

(8) $$\begin{array}{c} \Delta x_{\text{com}}=h_{\text{com}}\times \sin\theta_{p},h_{a,\text{com}}=h_{\text{com}}\times \cos\theta_{p}, \end{array}$$

where *h*_com_ and *h*_*a*,com_ are the body height on the flat ground and the adaptive height on the slope.

When walking on the slope, it is necessary to adjust the swing foot trajectory in an appropriate manner. As shown in Figure 6, the planned foot trajectory on flat ground was first rotated toward the slope (from the green dotted line to the gray dotted line). However, the trunk position relative to the foot adapted to the slope and the previous foot planning did not match the current state, which led to unsatisfactory movement. To achieve matching in real time, the motion trajectory of the swing leg was adjusted based on the corresponding CoM position, and the highest point of the swing leg was calculated as follows:

(9) $$\begin{array}{c} \Delta x_{sf}=h_{sf}\times \sin\theta_{p},h_{a,sf}=h_{sf}\times \cos\theta_{p}, \end{array}$$

where *h*_*sf*_ is the highest point of swing leg on the flat, *h*_*a*,*sf*_ is the adaptive highest point on the slope ground, and Δ*x*_*sf*_ is the compensation offset of the swing leg in forward direction. In the flight process, the target position of the foot is calculated by cubic spline interpolation, which can achieve both a starting and ending velocity acceleration equal to zero and ensure more stable movement.

## 5. Simulation and Experiments Results

### 5.1. Simulation

**Figure 7:**
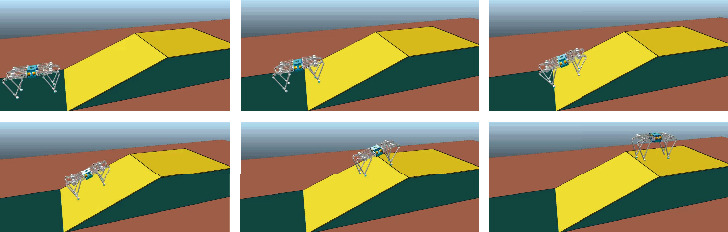
Simulation snapshots of the up-slope.

This section discusses the simulations of robot's locomotion on uneven terrain with the proposed method. In this study, the V-REP dynamic software and MATLAB were used to conduct simulations. To verify the proposed terrain estimation method and the corresponding adaptive motion strategy for the quadruped robot, a path starting from the flat ground and then gradually moving to the slope with trot gait was set. In this process, the space plane occupied by the robot's feet is constantly changing.  Figure 7 shows the snapshots of simulation.

**Figure 8:**
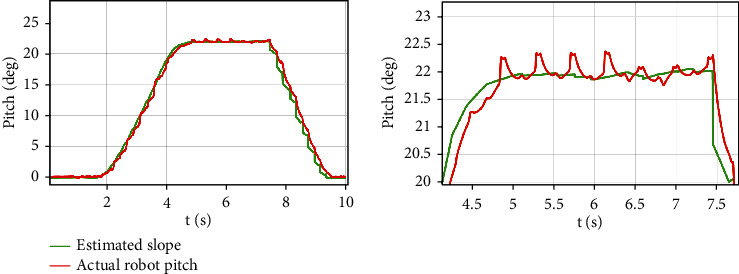
The tracking curve during uphill motion based on adaptive terrain.

Figure 8 shows the result of simulation, walking on the flat ground occurred from 0 s to 1.2 s, and the adjustment stage of gradual transition from the horizontal plane (0Â°) to the preset slope angle (22Â°) occurred from 1.2 s to 4.2 s. In this movement stage, the fore legs are on the rising slope, and the hind legs are on the flat ground. We can find that the angle of virtual slope based on the unknown estimation of foot tip increases linearly, which is consistent with our uniform motion. The stage wherein the robot was completely on the 22Â° slope was from 4.2 s to 7.5 s. The partially enlarged view of the slope angle tracking during the stage of movement on the slope is shown in Figure 8. As can be seen, the slope angle error between the evaluation and preset fluctuated in the range of -0.08Â° to 0.03Â°, which confirms the accuracy of the proposed slope angle evaluation method. Moreover, the actual pitch angle is higher by approximately 0.2Â° compared with the target value. The reasons for this may be that the controller parameters are not ideal and that the offline CoM position estimation of the robot is not accurate. These issues will be improved in future work.

### 5.2. Experiments

**Figure 9:**
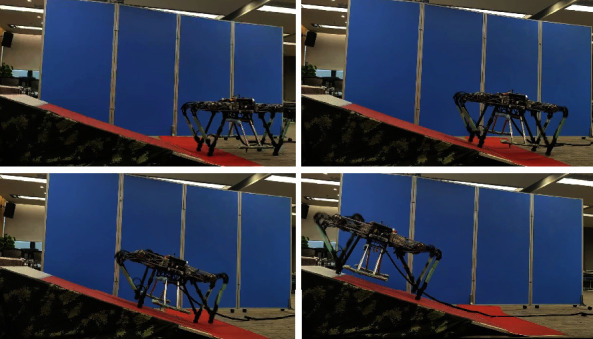
Snapshots of the up-slope experiment.

**Figure 10:**
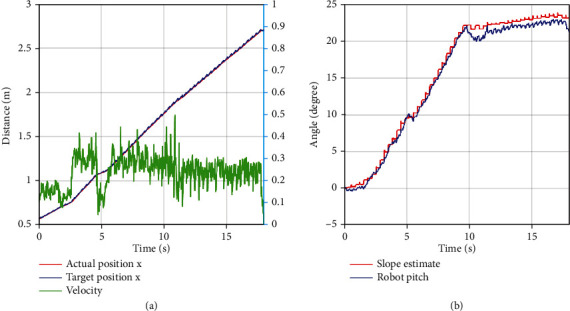
Up-slope experiment data. (a) Left plot is the position information; (b) right plot is the slope angle estimate by our method and robot body pitch angle measured by IMU.

**Figure 11:**
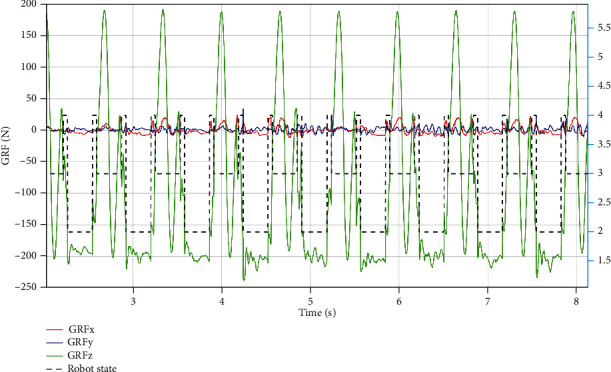
Up-slope experiment data of front left leg. The red, blue, and green lines present the GRF; black dotted line is the robot state. The values of 1, 2, 3, and 4 are flag variables which can be got by the state machine based on the combine of event-based (force sensor) and time-based (planning). We divided the robot state into 4 states: four leg supporting phase, two double leg supporting phase, and air phase. The value of 4 denotes the four-leg supporting phase, 2 and 3 denote diagonal double leg supporting phase, and 1 denotes air phase.

**Figure 12:**
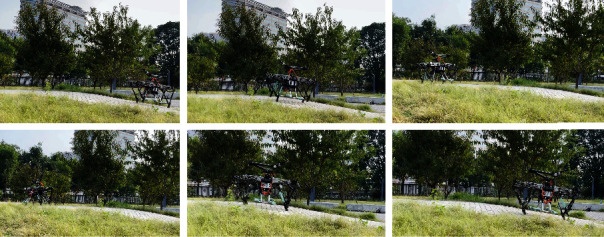
Snapshots of the up and down-slope experiment in outdoor.

**Figure 13:**
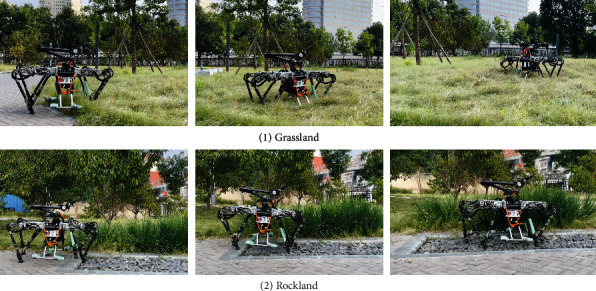
Snapshots of the grassland and rockland experiment.

**Figure 14:**
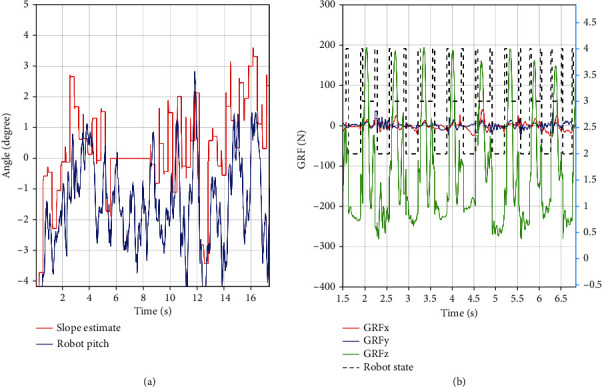
Rock land experiment data. (a) Left plot is the slope angle and robot body pitch; (b) right plot is the foot force and robot state.

Indoor and outdoor experiments were conducted to validate the effectiveness of the proposed method. First, the up-slope experiments were conducted on a 22Â° slope setup indoor.  Figure 9 shows the sequential snapshots captured on the up-slope with a trot gait. As shown in Figure 10, the position tracking is satisfactory, and only a small position error existed, owing to the IMU error. The estimation of the slope condition is ideal, and certain fluctuation existed because the slope parameter was estimated in the four-leg supporting phase. The blue line indicates the data obtained from the IMU after Kalman filtering and coincides with the slope parameter. There was a larger error of the robot pitch angle when the back leg located on the slope, because the step between slope and ground may result in a slide. The green line indicates the actual velocity data obtained from the IMU, the robot walked from a plat ground to the slope before 10 s, and the velocity had a larger fluctuation in this transfer process.  Figure 11 shows the robot state and GRF of one leg. The vertical GRF fluctuates around the 200 N at stance phase; the period smooth curve presents the force at swing phase, which confirms that the robot state machine is stable and reliable. To keep a stable trot gait, there was a short four-leg supporting phase. Obviously, there is no slide on the slope because the GRF values of horizontal are small enough. Finally, to validate the robust locomotion of our quadruped robot, three outdoor experiments were performed with an arm functioning as disturbance. In this paper, the arm in these experiments was inactivated which can be as a mass model. Figures 12 and 13 show the sequential snapshots captured on the slope, grassland, and rock terrain, respectively.  Figure 14 shows the result data of rock terrain; the estimated slope and robot pitch angle have a larger fluctuation compared with the flat slope condition. Also, there is a slide on this ground because the cobblestone is slippery, and the foot forces are fluctuant consequentially.  Table 1 lists the main locomotion parameters of the robot in the outdoor experiment. The weights of the foot force distribution should be changed to adapt to different terrain.

## 6. Conclusions

Dynamic locomotion and adaptability to complex terrain are important indicators of quadruped robot performance. By the comparative analysis of different structures, this study designed a symmetrical parallel quadruped robot with the advantages of strong force production and excellent motion performance. Then, a control framework based on VMC was established to enhance the robot's ability of adapting to unknown complex terrain. This framework mainly involves the optimal allocation of GRFs based on QP and a complete terrain adaptation method. The proposed terrain adaptation method uses spatial support plane estimation based on the generalized least squares method and fuses the body orientation information and joint encoder information without additional perceptual or visual information to realize the balance control of the robot by adapting the trunk posture and swing leg trajectory, respectively. This method is robust to the drift of the robot attitude measurement, and the involved calculations are easy. The simulation and experimental results obtained for indoor and outdoor locomotion reveal that the robot has strong adaptive ability on uneven terrain and can resist certain external impact, which demonstrates the effectiveness and robustness of the proposed method. In a future work, we plan to improve the highly dynamic locomotion, such as running and jumping of the quadruped robot.

## Figures and Tables

**Table 1:** Experiment parameter.

Parameter	Symbol	Value	Units
Body height	H	0.55	m
Foot height of swing leg	h	0.07	m
Force proportional gain of body	*K*_*P*_^*B*^	[350,1400,2500,1000,550,280]^*T*^	N/m
Force derivative gain of body	*K*_*d*_^*B*^	[20,30,100,30,30,28]^*T*^	Ns/m
Force proportional gain of leg	*K*_*P*_^*L*^	[150,300,2200]^*T*^	N/m
Force derivative gain of leg	*K*_*d*_^*L*^	[12,15,200]^*T*^	Ns/m
Weights for foot force distribution	S	diag(0.7,0.5,0.7,2.0,1.5,0.3)	
Weights for reducing joint torques	W	diag(0.001⋯)	

## Data Availability

The data used to support the findings of this study are available from the corresponding author upon request.
